# The development and validation of a medicines optimisation tool to protect the physical health of people with severe mental illness (OPTIMISE)

**DOI:** 10.1186/s12888-022-04235-0

**Published:** 2022-09-03

**Authors:** Aoife Carolan, Dolores Keating, Stephen McWilliams, Caroline Hynes, Mary O’Neill, Fiona Boland, Sharon Holland, Judith Strawbridge, Cristín Ryan

**Affiliations:** 1 Saint John of God Hospital, Stillorgan, Co. Dublin Ireland; 2 School of Pharmacy and Biomolecular Science, Royal College of Surgeons Ireland, 123 St Stephen’s Green, Dublin 2, Dublin, Ireland; 3 School of Medicine and Medical Sciences, University College Dublin, Belfield, Dublin 4, Ireland; 4 Tallaght University Hospital, Dublin 24, Ireland; 5 Data Science Centre, Royal College of Surgeons in Ireland, 123 St Stephens Green, Dublin 2, Dublin, Ireland; 6 Northumberland, Tyne and Wear NHS Foundation Trust, Newcastle upon Tyne, UK; 7 School of Pharmacy and Pharmaceutical Sciences, Trinity College Dublin 2, Dublin, Ireland

**Keywords:** Mental Illness, Medicines Optimisation, Physical Health, Screening Tool, Severe Mental Illness

## Abstract

**Background:**

The life expectancy of people with severe mental illness (SMI) is shorter than those without SMI, with multimorbidity and poorer physical health contributing to health inequality. Screening tools could potentially assist the optimisation of medicines to protect the physical health of people with SMI. The aim of our research was to design and validate a medicines optimisation tool (OPTIMISE) to help clinicians to optimise physical health in people with SMI.

**Methods:**

A review of existing published guidelines, PubMed and Medline was carried out. Literature was examined for medicines optimisation recommendations and also for reference to the management of physical illness in people with mental illness. Potential indicators were grouped according to physiological system. A multidisciplinary team with expertise in mental health and the development of screening tools agreed that 83 indicators should be included in the first draft of OPTIMISE. The Delphi consensus technique was used to develop and validate the contents. A 17-member multidisciplinary panel of experts from the UK and Ireland completed 2 rounds of Delphi consensus, rating their level of agreement to 83 prescribing indicators using a 5-point Likert scale. Indicators were accepted for inclusion in the OPTIMISE tool after achieving a median score of 1 or 2, where 1 indicated strongly agree and 2 indicated agree, and 75^th^ centile value of ≤ 2. Interrater reliability was assessed among 4 clinicians across 20 datasets and the chance corrected level of agreement (kappa) was calculated. The kappa statistic was interpreted as poor if 0.2 or less, fair if 0.21–0.4, moderate if 0.41–0.6, substantial if 0.61–0.8, and good if 0.81–1.0.

**Results:**

Consensus was achieved after 2 rounds of Delphi for 62 prescribing indicators where 53 indicators were accepted after round 1 and a further 9 indicators were accepted after round 2. Interrater reliability of OPTIMISE between physicians and pharmacists indicated a substantial level of agreement with a kappa statistic of 0.75.

**Conclusions:**

OPTIMISE is a 62 indicator medicines optimisation tool designed to assist decision making in those treating adults with SMI. It was developed using a Delphi consensus methodology and interrater reliability is substantial. OPTIMISE has the potential to improve medicines optimisation by ensuring preventative medicines are considered when clinically indicated. Further research involving the implementation of OPTIMISE is required to demonstrate its true benefit.

**Trial registration:**

This article does not report the results of a health care intervention on human participants.

**Supplementary Information:**

The online version contains supplementary material available at 10.1186/s12888-022-04235-0.

## Background

People with severe mental illness (SMI), including schizophrenia, schizoaffective disorder, bipolar illness and severe affective disorder have a life expectancy up to 30 years shorter than those without SMI [1–4]. Pooled data estimate that all-cause mortality is more than doubled for people with any mental illness (relative risk 2.22 from a meta-analysis of 148 studies) [5]. Death by suicide is an important contributing factor for premature death of people with SMI, however, much of the health inequality is attributed to modifiable risk factors, an increased prevalence of physical illness and multimorbidity (the presence of two or more long-term health conditions), adverse effects of psychotropic medicines and deficits in access to, or utilisation of, health care services. [1, 2, 5–8].

Mortality rates for chronic physical diseases in people with SMI are higher than would be expected when compared with the general population suggesting sub-optimal management of co-morbidities [9–11]. Inequalities exist with respect to access to interventions for primary prevention. People with SMI are less likely to have regular contact with a general practitioner or receive routine physical health screening, monitoring or interventions than the general population [1, 2, 4]. System barriers include fragmentation and separation of mental health services from other medical services. There is some evidence to indicate reduced mortality with integrated care [4, 9, 12]. All of these factors can lead to delays in the diagnosis of physical illness as well as poorer management.

The value of psychotropic medicines in improving life expectancy and health related quality of life is well recognised. Poor adherence to psychotropic medicines for SMI not only increases the risk of relapse but also increases hospitalisation and reduces quality of life [13, 14]. Psychotropic medicines are however, associated with significant risk of errors and adverse drug events (ADEs), increasing the risk of co-morbidities [15, 16]. Studies in Australia have shown that over 80% of people with a psychotic illness experience side effects from their medicines [17, 18]. Reports suggest that there is often a focus on symptom management for SMI and clinicians pay less attention to side effects of medicines and the potential impact these may have on quality of life and functioning [18].

Adverse effects experienced by people with SMI include cardiovascular disease, weight gain, metabolic syndrome (MetS), Type 2 diabetes, thyroid disease and respiratory illness. Atypical antipsychotic agents cause weight gain in 15–72% of patients [1]. Obesity is an independent risk factor for MetS which confers a fourfold increased risk of diabetes and a two-fold increased risk of coronary heart disease, stroke and premature mortality [19]. Despite this, primary prevention for chronic illness is often overlooked in people with SMI [16].

A systematic review of treatment guidelines for first episode schizophrenia highlights that studies have failed to demonstrate superiority for any individual antipsychotic agent [20]. Medicines to treat SMI should be selected based on efficacy, relative adverse effect profiles, past medical history and risk factors for chronic illness. Medicines need to be optimised to ensure adequate control of symptoms of mental illness while minimising negative physical health outcomes.

There are people with SMI for whom high dose antipsychotic therapy or antipsychotic polypharmacy is necessary [21, 22]. Hence, there is a requirement for alternative strategies to manage adverse effects other than dose reduction or avoidance of antipsychotic polypharmacy. The co-prescribing of medicines that could potentially ameliorate psychotropic related adverse effects, if a switch in psychotropic agent is not possible or an equally effective alternative is not available, should be considered as part of a medicines optimisation strategy.

Lifestyle behaviours associated with SMI contribute to modifiable risk factors and increased prevalence of physical illness. One example is the prevalence of smoking and smoking-related illness amongst people with SMI. People with schizophrenia are three times more likely to smoke than the wider population [22–24]. Additionally, approximately 42% of all cigarettes smoked by the English population are smoked by people with a mental illness [9]. This lifestyle behaviour, known to significantly increase the risk of mortality and cardiovascular risk, is significantly more prevalent among people with SMI and yet interventions to reduce this risk are not equally or effectively targeted at this group of people [23]. Similar inequalities are observed among other lifestyle behaviours such as diet, exercise, obesity and substance misuse [1, 4, 25].

Multimorbidity and the complexities associated with managing co-morbid illness is becoming increasingly recognised, not least among people with SMI.[6, 26–28] Clinical guidelines are largely created for individual disease states and high quality randomised controlled trials often exclude multimorbidity [4]. The fragmented nature of healthcare systems, where physical and mental health care is divided, emphasises the need to support clinicians who treat people with SMI in managing co-morbidities and optimising their physical health needs [4, 9, 29].

Diagnostic overshadowing, the concept of healthcare staff incorrectly attributing symptoms of physical ill health to a mental health condition [30], is referred to in a recent population-based cohort study of end-of-life care among people with schizophrenia and cancer. People with schizophrenia were more likely to die earlier, have shorter durations from cancer diagnosis to death and have co-morbidities. The authors suggest that diagnostic overshadowing may contribute to this finding in that physical symptoms are misattributed to mental illness leading to delayed screening, diagnosis and treatment, and an increased likelihood of a terminal diagnosis. This was further explained by the fact that people with schizophrenia had a lower frequency of imaging examinations in the last month of life than the control group [31]. Support is therefore needed for those treating SMI to remove barriers to screening and intervening for physical health co-morbidities [32].

The lack of high quality randomised controlled trials assessing interventions to protect the physical health of people with SMI means that there is a reliance on expert opinion and extrapolation of evidence from trials in healthier patients. Guidance on the appropriate prescribing of psychotropic medicines for people with SMI is provided by the recognised bodies such as the British Association of Psychopharmacology (BAP), the American Psychiatric Association (APA) and the World Federation of Societies of Biological Psychiatry (WFSBP) in systematic reviews and consensus statements. The BAP guidance on the management of weight gain, metabolic disturbances and cardiovascular risk associated with psychosis and antipsychotic drug treatment and the NICE adapted Lester UK Positive Cardiometabolic Health Resource Tool encourage clinicians to actively monitor and intervene to protect the physical health of people with SMI [25, 33]. Whilst guidelines are welcome, there is little in the way of quick reference guidance to assist clinicians at the point of prescribing in the identification of prescribing omissions and the management of multimorbidity in people with SMI. In their current format, the guidelines do not facilitate optimising the use of medicines for primary and secondary prevention of physical illness.

Medicines optimisation is a person centred approach aimed at ensuring the safe and effective use of medicines such that individuals obtain the best outcomes from their medicines [34]. Screening tools are commonly incorporated into clinical practice to improve medicines optimisation. These tools often incorporate a list of prescribing criteria to guide clinicians in the review of medication. Published screening tools target various patient populations. For example, PROMPT is a tool targeting middle-aged adults with multimorbidities, STOPP/START and Beers’ Criteria target adults over 65 years, STOPPFrail targets frail adults with a shorter life expectancy and PIPc targets paediatrics [35–39]. The STOPP/START tool assists the identification of potentially inappropriate medicines (PIMs) and potential prescribing omissions (PPOs). PPO is the failure to prescribe an indicated medicine, despite the lack of contra-indications to this medicine [40]. The application of STOPP/START in practice has demonstrated improvements in prescribing and patient outcomes. Specifically, reductions in medication related hospitalisation, healthcare resource utilisation, and cost can be achieved with the implementation of these screening tools [41, 42].

Randomised controlled trials evaluating technology-based medicines optimisation interventions such as PINCER and the on-going SENATOR trial are evaluating their effectiveness in reducing medication errors and healthcare costs.[43, 44] A NICE approved IT-based intervention aimed at reducing potentially inappropriate prescribing [the PINCER intervention] was implemented across 2432 GP practices in England. Follow-up data from 1060 GP practices, showed that implementation of the PINCER intervention resulted in a reduction in instances of potentially inappropriate prescribing from 92,762 at baseline to 79,375 instances 6 months after the intervention [45]. SMASH, an enhanced PINCER intervention, demonstrated reduced rates of potentially inappropriate prescribing which was sustained for up to 1 year after the intervention [46].

Medicines optimisation screening tools can contain indicators that are relevant to mental illness and psychotropic medicines, however, there is no specific emphasis in these tools for screening and intervening to improve physical health outcomes in SMI. There is a need for a validated screening tool that targets the tailored monitoring and management of physical health in people with SMI.

Khawagi et al. published a systematic review in 2019 identifying potential prescribing safety indicators related to mental health disorders and medications. This review identified 245 potential prescribing safety indicators of which, only 5 related to PPOs in mental illness [47]. Evidence from UK mental health hospitals indicates a high prevalence of PPOs among people with mental illness [48]. There is, therefore, a need to develop prescribing guidance to facilitate medicines optimisation for people with SMI.

Medicines optimisation is a complex multifaceted intervention and screening tools are unlikely to cover all aspects. A screening tool could, however, aid clinicians in prompting consideration for PPOs and assist clinicians in overcoming barriers such as diagnostic overshadowing. A user friendly screening tool should lead to an overall improvement in prescribing quality, prevent avoidable ADEs and medication related hospitalisations, improve patient adherence, satisfaction with prescribed treatment and quality of life [32].

The aim of this research was to design a medicines optimisation tool (OPTIMISE) to assist healthcare professionals who provide care for people with SMI, including doctors and pharmacists, in optimising medicines to protect the physical health of adults with SMI.

## Methods

### Study design

Delphi Consensus methodology was used to develop the OPTIMISE tool. This methodology has been successfully used in the development of other screening tools *e.g.* STOPP/START, PROMPT. Statistical analyses methodology similar to that used to develop and validate other screening tools was adopted in the development and validation of OPTIMISE. Ethical approval was granted by the Research and Ethics Committees at Saint John of God Hospital, Dublin and the Royal College of Surgeons in Ireland, Dublin.

### Development of the OPTIMISE prescribing indicators

A literature review was undertaken to explore the potential initial content of OPTIMISE. Literature was reviewed to identify monitoring recommendation and treatment interventions relevant to people with SMI including lifestyle interventions and/or pharmacological interventions. A broad search string [Severe mental illness OR Schizophrenia OR Psychosis OR Bipolar OR Depression] AND [Prescribing Omissions OR Potentially Inappropriate Prescribing OR Medicines Optimisation OR Medication Omission OR Prescribing Tool] AND [Interventions OR Treatment] was used to search Medline and PubMed databases. The search string and literature search were reviewed internally by the academic pharmacists on the research team. Existing published guidelines and reference texts identified by the research team, based on their clinical and academic experience, were reviewed for evidence of primary and secondary prevention prescribing strategies of relevance to people with SMI. These guidelines included British Association of Psychopharmacology, European Society of Cardiology, World Federation of Societies of Biological Psychiatry, American Psychiatry Association, National Institute for Health and Care Excellence and Scottish Intercollegiate Guidelines Network guidelines.

A list of indicators was developed from the recommendations in the literature and grouped according to physiological system. The list was reviewed by clinical members of the research team, including academic pharmacists, experts in methodological components of prescribing tool development, experts in mental health pharmacy, psychiatry and primary care. Using the evidence from the literature search, the team considered the relevance of each recommendation and its inclusion in the initial draft of OPTIMISE. Some indicators were modified to account for additional risks in people with SMI as guided by the evidence. For example, it is recommended that women who are pregnant or planning a pregnancy and are prescribed an antiepileptic medicine should be prescribed folic acid 5mg daily. This was incorporated into an indicator in the OPTIMISE tool and modified to include the warning that folic acid may reduce the efficacy of lamotrigine and the recommendation to monitor the woman for deterioration in mental health. As such, the modified indicator combined two separate evidence-based recommendations. Following review, the initial OPTIMISE tool included 83 indicators. This list was transferred into a web-based platform (survey monkey), to obtain a consensus agreement on each criterion.

### Delphi validation

A total of 27 experts were invited to take part in a Delphi panel to develop and validate OPTIMISE. The specialists were recognised as experts in their fields and selected by the research team, based on their clinical experience in the areas of general medicine or mental health, academic credentials and geographical diversity (UK and Ireland). The experts included a combination of pharmacists and medical doctors. Following two reminders, sent two weeks apart, 17 experts agreed to take part. The panel was presented with 83 prescribing indicators, each in the format of a factual statement, a Likert scale to note level of agreement and an optional section for commentary for each statement (Table 1).

**Table 1:** Example of a prescribing indicator in questionnaire format provided to the Delphi panel

**Section A: Cardiovascular System** **Indicator: Overweight/Obesity**	1 = Strongly Agree, 5 = Strongly Disagree					
	1	2	3	4	5	Comments
Lifestyle interventions should always be part of the first line approach to reducing BMI in overweight/obese* individuals with SMI (*WHO defines overweight as BMI ≥ 25 kg/m^2^ and obesity as BMI ≥ 30 kg/m^2^)						
Lifestyle interventions should be continued alongside additional interventions to reduce BMI in overweight /obese individuals with SMI						
Consider adjunctive metformin, unless contraindicated, in people with SMI who are prescribed clozapine or olanzapine and who have demonstrated early weight gain* *early weight gain is defined as 5% of body weight in the first month of treatment and is a predictor of long-term weight gain (> 15% over 3 months)						

Consenting panellists completed two rounds of the Delphi process between April and October 2019.

Indicators with a median value of 1 or 2 and a 75th centile value of ≤ 2 were accepted for inclusion in the OPTIMISE tool. Indicators with a median value > 2 were rejected. Where the median value was 1 or 2 and the 75^th^ centile value was > 2, the indicator was reviewed by the research team. The comments noted were reviewed by the research team and the statements revised based on this feedback provided. The revised indicators were then included in the next round of Delphi validation.

In round two, panellists were given a summary of the results for round 1. This included a list of accepted indicators from round 1, a list of rejected indicators from round 1 and a list of indicators where consensus had not been achieved. Round 1 feedback was presented in a separate word document and distributed to individual Delphi panellists via email. The median Likert values and inter-quartile range for each of the indicators were also included. Panellists were then asked to review the remaining indicators where consensus was not achieved, in light of feedback from round one. Panellists were given four weeks to respond to each round and reminder emails were circulated to panellist on weeks 2 and 3 of each round.

Statistical analyses were performed using STATA 16 SE [35, 37, 41]. In both rounds, the median Likert response, inter-quartile range and 75^th^ centile values were calculated for each indicator.

### Interrater reliability

Once validated using Delphi methodology, interrater reliability (IRR) of OPTIMISE was explored. Twenty-one datasets were prepared from prospectively recruited inpatients across two wards in an Irish psychiatric teaching hospital. Each dataset represented an individual patient. To recruit these patients, ward lists were reviewed and every second patient was selected from the list. Patients received an information leaflet and explicit informed consent was obtained to include their anonymised data in datasets for the IRR exercise. Each dataset contained the following clinical details: age and gender, co-morbidities, relevant blood results and all current prescribed medication. Additional clinical information was provided including Body Mass Index (BMI), alcohol intake, smoking status and estimated cardiovascular risk using QRISK3 [59]. In preparing the datasets, if the research team identified omissions in the routine physical health monitoring requirements, this was highlighted to the treating physician via a gatekeeper on the research team to maintain the pseudonymity of the datasets. One dataset was randomly selected from the 21 datasets using a random selection generator on Microsoft Excel version 14.0 (Microsoft Corporation, 2010 Redmond, WA 8052–6399. United States). This was used as a sample dataset for demonstration purposes, and the remaining twenty were used for the IRR study.

Two clinicians, one pharmacist and one physician, both with extensive experience in mental health practice and familiar with the tool through involvement with the research team or Delphi panel reviewed the 20 datasets independently. They then discussed the application of OPTIMISE in detail until full agreement was reached and a set of standard recommendations were agreed. These recommendations were used as the standard recommendations against which responses from IRR study participants were compared. IRR study participants included two pharmacists and two physicians with at least 3 years’ experience in mental health and who contributed to the Delphi panel.

A booklet was provided to study participants which included a cover letter with detailed instructions on how to apply OPTIMISE in a clinical setting, the 20 datasets, a copy of the OPTIMISE tool and one example case. Each of the four participants applied the OPTIMISE tool independently to the same cohort of 20 datasets. Participants were invited to provide comments on the criteria used for each case. Participants were also asked to record the time taken to apply the OPTIMISE tool to each patient dataset. The results were compared to the standard recommendations already agreed to establish IRR.

Data were analysed using STATA 16 SE. Cohen’s kappa coefficient, the Ƙ statistic (i.e. chance corrected measure of agreement) was calculated for comparison to establish level of agreement between pharmacists and physicians. The Ƙ statistic was interpreted as poor if 0.2 or less, fair if 0.2–0.4, moderate if 0.41–0.6, substantial if 0.61–0.8 and good if 0.81–1.0 [35, 49, 50]. Previous IRR studies applied to the START tool (version 1) have demonstrated Ƙ values ranging from 0.68 when tested between two raters to 0.9 for larger groups of specialists [35, 50].

Proportion of positive agreement (ppos) and proportion of negative agreement (pneg) was also established, to determine the level of agreement and consistency of agreement between pharmacists and physicians in their decision to apply or not apply the indicators to the datasets [50, 51].

## Results

OPTIMISE was drafted with reference to the literature. Eighty-three indicators were drafted and grouped according to physiological system including CV system (*n* = 23), endocrine (*n* = 16), gastro-intestinal (*n* = 2), blood and nutrition (*n* = 9), respiratory (*n* = 12), musculoskeletal (*n* = 12) and lifestyle intervention (*n* = 9). A total of 17 experts consented to participate in the Delphi panel. The panel of 17 experts consisted of consultant psychiatrists (*n* = 5), senior academic pharmacists (*n* = 2), clinical pharmacists with interests in medicines optimisation (*n* = 2), specialist mental health pharmacists (*n* = 3), community pharmacist (*n* = 1), psychiatry registrars (*n* = 3) and an academic primary care physician (*n* = 1). Most of the panel were practicing in Ireland (*n* = 15) and two panellists were practicing in the UK (Scotland and England). Of the 17 experts, 16 were affiliated with university teaching hospitals across the UK or Ireland.

**Fig. 1:**
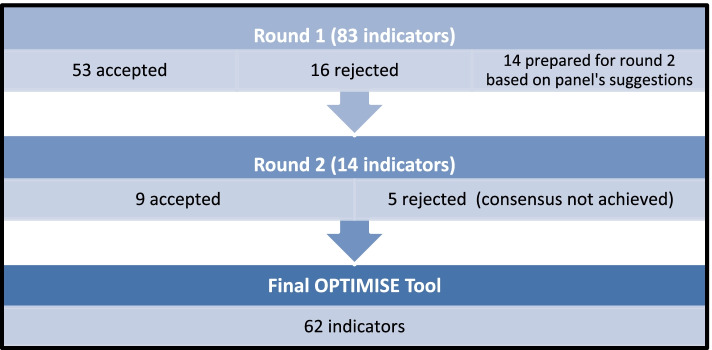
Flow chart demonstrating the Delphi Consensus Process

Delphi validation was completed in two rounds generating a final OPTIMISE tool of 62 indicators (Fig. 1). Round 1 was completed by all of the 17 panellists. Round 2 was completed by 16 of the 17 panellists. Of the initial 83 prescribing indicators, consensus was achieved for 69 in the first round where 53 indicators were accepted and 16 indicators were rejected. The 14 remaining indicators where consensus was not achieved were revised by the research team taking into account comments from the panellists and sent to the expert panel for a second round of Delphi validation.

In round 2, 9 indicators were accepted but consensus was not achieved for five indicators. These five indicators were reviewed by the research team and, following a review of the panellists’ additional comments, it was decided that consensus was unlikely to be achieved in a further round of Delphi. These indicators were not included in OPTIMISE.

### Statistical analysis

Table 2 shows the median value and 75^th^ centile value for the 53 prescribing indicators accepted in the first round of the Delphi validation exercise.

**Table 2:** Prescribing indicators accepted in round 1 of Delphi exercise with Median and 75^th^ Centile Value

	**Prescribing Indicator**	**Median Score**	**75**^**th**^** Centile Value**
1	Assess CV risk in all adults with SMI over 40 years using a validated cardiovascular risk assessment tool eg. SCORE, QRISK2, QRISK3 and review on a regular basis eg. annually	1	2
2	Consider statin therapy (eg. Atorvastatin 20 mg daily) for those adults who have ≥ 10% 10-year risk of developing CV disease using a validated CV risk assessment tool	2	2
3	Commence high intensity statin therapy (eg. atorvastatin 80 mg daily) in adults with existing CV disease for secondary prevention. Lower doses can be chosen if there are potential drug interactions, high risk of adverse effects or patient preference	2	2
4	Commence statin therapy (eg. atorvastatin 20 mg daily) for the primary and secondary prevention of CVD in adults with chronic kidney disease (CKD). Discuss the use of higher doses with a specialist in nephrology/cardiology if eGFR < 30 mL/min	2	2
5	If total cholesterol > 9 mmol/L, non-HDL > 7.5 mmol/L or triglycerides (TG) > 10 mmol/L refer to a metabolic specialist	2	2
6	Statins with a high degree of lipophilicity eg. simvastatin may be associated with central nervous system disturbance eg. sleep disturbance, nightmares. Consider more hydrophilic statins eg. atorvastatin, pravastatin in people with SMI	2	2
7	Commence antihypertensive therapy in adults < 80 years with a clinic blood pressure of ≥ 140 mmHg systolic and/or ≥ 90 mmHg diastolic (stage 1 hypertension) and subsequent ambulatory blood pressure monitoring (ABPM) daytime average ≥ 135/85 mmHg who have one or more of the following: i.Target organ damage ii.Established CV disease iii.Renal disease iv.Diabetes (Type 1 or 2) v.A 10 year CVD risk of ≥ 20%	2	2
8	Commence antihypertensive therapy to adults of any age with clinic blood pressure ≥ 160/100 mmHg and ABPM ≥ 150/95 mmHg (stage 2 hypertension)	1.5	2
9	Lifestyle interventions should always be part of the first line approach to reducing BMI in overweight/obese individuals with SMI	1	2
10	Lifestyle interventions should be continued alongside additional interventions to reduce BMI in overweight/obese individuals with SMI	1	1.75
11	Offer smoking cessation advice to all people with SMI who smoke.(See section E1 for full smoking cessation intervention)	1	1
12	Commence antiplatelet therapy (aspirin or clopidogrel or prasugrel or ticagrelor) in adults with SMI with a documented history of coronary, cerebral or peripheral vascular disease	2	2
13	For people who present with catatonia, consider the impact of this on mobility when assessing VTE risk and reassess where appropriate	2	2
14	Prescribe pharmacological VTE prophylaxis with low molecular weight heparin following risk assessment according to local or national clinical guidelines	2	2
	If glycosylated haemoglobin (HbA1c) 42–47 mmol/mol and fasting plasma glucose (FG) 5.5–6.9 mmol/L:		
15	i.offer an intensive structured lifestyle education programme	1	2
16	ii.if ineffective, consider a trial of metformin	2	2
17	In existing diabetes, if HbA1c ≥ 48 mmol/mol or FG ≥ 7.0 mmol/L refer to an endocrine specialist for optimisation of diabetic control except in older adults where a HbA1c upper limit of 58 mmol/mol is acceptable	1	2
18	Consider thyroid supplementation with levothyroxine in adults with SMI if thyroid stimulating hormone (TSH) > 10 mU/L	2	2
19	When starting levothyroxine, 50-100mcg daily is the recommended starting dose for most adults. In adults over 65 years or adults with ischaemic heart disease lower doses of 25mcg daily can be initiated	2	2
20	TFTs should be performed 4–6 weeks after starting levothyroxine and dose adjusted according to response. TSH is the most reliable marker of adequacy of replacement of treatment and a value within the reference range 0.4–4.0mIU/L) should be considered the therapeutic target	2	2
21	Adults with subclinical hypothyroidism (ie. TSH elevated but < 10mIU/L and T4 within normal range) should have their TFTs repeated within 3–6 months to exclude transient causes of elevated TSH. The measurement of thyroid antibodies in subjects with subclinical hypothyroidism helps to establish the risk of developing overt hypothyroidism	2	2
22	Do not routinely start thyroid supplementation with levothyroxine for the management of depression in a euthyroid adult or an adult with symptoms that overlap those of hypothyroidism	2	2
23	Adults with SMI with evidence of hyperthyroidism/thyrotoxicosis on blood results should be referred to an endocrine specialist	1	1
24	Monitor baseline prolactin for all people with SMI before initiating an antipsychotic known to raise prolactin	1	2
25	Systematically assess for symptoms of hyperprolactinaemia in people with SMI who are prescribed an antipsychotic at 3 months and biannually thereafter	2	2
26	If symptoms of hyperprolactinaemia appear in a person taking an antipsychotic at any stage, measure prolactin levels	2	2
27	In symptomatic hyperprolactinaemia, where a dose reduction or a switch to an alternative antipsychotic with a lower potential to elevate prolactin is not possible, consider adjunctive aripiprazole at a dose of 5 mg daily. Repeat prolactin levels after at least 1 week to establish benefit	1	2
28	Where prolactin levels are > 3000mIU/L, refer to an endocrine specialist to rule out other causes of elevated prolactin	2	2
29	Dopamine agonists (eg. cabergoline, bromocriptine) should not be initiated in adults with SMI for the management of hyperprolactinaemia except under specialist endocrine advice due to the risk of psychosis	1	2
30	Consider the increased risk of bleed (not limited to gastrointestinal bleed) when a selective serotonin reuptake inhibitor (SSRI), serotonin noradrenaline reuptake inhibitor (SNRI) or tricyclic antidepressant (TCA) is combined with aspirin, non-steroidal anti-inflammatory drugs (NSAIDs) and direct oral anticoagulants (DOACs)	1	2
31	Identify and manage anaemia as for the general population	1	2
32	Monitor folic acid levels periodically in people who are prescribed antiepileptic drugs and correct any folic acid deficiencies	2	2
33	Always identify and correct vitamin B12 deficiency before prescribing folic acid	1	2
34	Consider folic acid 5 mg daily for women who are pregnant or planning pregnancy and who are taking antiepileptic drugs including valproate, carbamazepine and possibly lamotrigine. Note that folic acid may reduce the efficacy of lamotrigine and monitor for deterioration in mental illness	1	2
35	Do not routinely prescribe folic acid as an augmenting agent in the treatment of depression	2	2
36	Identify and manage vitamin B12 deficiency as for the general population	2	2
37	Do not routinely prescribe Omega 3 fish oils in people with SMI	2	2
38	Ask and document smoking status for all patients with SMI	1	1
39	Opportunistically offer smoking cessation advice to all people with SMI who smoke documenting this advice and current readiness to quit eg. ‘not interested in quitting’, ‘not right time’, ‘would like to but not ready’ etc. For those not ready, check-in again on future interactions	1	1
40	For those ready to make a quit attempt consider nicotine replacement therapy (NRT), varenicline or buproprion to support smoking cessation for people who smoke more than 10 cigarettes per day or who smoke within 30–60 min of waking with consideration for contraindications and comorbidities	2	2
	When selecting a pharmacological intervention, consider the following:		
41	i.Combinations of different forms of NRT can be used	2	2
	ii.Tobacco smoking can alter the pharmacokinetics of psychotropic medications:		
42	Consider a dose reduction by up to 50% in patients taking clozapine who stop smoking abruptly. Carefully monitor for adverse effects of clozapine and destabilisation of mental illness. Perform a clozapine assay 3–5 days after dose adjustment or abrupt cessation of smoking	2	2
43	Consider a dose reduction by up to 20% in patients taking olanzapine who stop smoking abruptly. Carefully monitor for adverse effects of olanzapine and destabilisation of mental illness	2	2
44	Follow the most recent NICE/SIGN/GOLD/BTS guidance for the optimisation of medicines in COPD or Asthma	2	2
45	When managing an acute exacerbation of COPD or asthma in adults with SMI consider the potential for steroid induced mania/psychosis. Current guidance recommends prednisolone 40 mg/day for 5 days. A lower dose of 30 mg/day for the shortest possible duration may be warranted in adults with a history of psychosis/mania depending on the severity of the exacerbation	2	2
46	Inform adults with SMI who are prescribed systemic corticosteroid therapy of the risk of mania/psychosis at the point of prescribing and monitor for signs of mania/psychosis	1	1.25
	Carry out a fracture risk assessment using a validated tool such as QFracture or Frax to determine the need for antiosteoporosis therapy for:		
47	i.Women aged ≥ 65 years and men aged ≥ 75 years	2	2
48	ii.Adults over 50 years of age with risk factors [eg. body mass index < 18.5 kg.m2, history of falls, family history of hip fracture, oral steroid use (prednisolone > 7.5 mg/day or equivalent for ≥ 3 months, secondary causes of osteoporosis, smoking, > 14 units alcohol/week (women) or > 21 units alcohol/week (men)]	2	2
49	Adults > 50 years of age with a history of vertebral fracture should be considered for antiosteoporosis therapy with an oral bisphosphonate without necessarily requiring risk assessment or DEXA scan	2	2
50	Offer access to a combined health eating and physical activity programme to aid in the prevention of weight gain to all adults with psychosis or schizophrenia	1	1
	Advise all adults aged 19–64 years to aim to achieve one of the following each week:		
51	i.A mixture of moderate and vigorous aerobic activity ever week and strength exercise on 2 or more days that work all of the major muscles	2	2
52	Adequate dietary calcium consumption is recommended to meet reference intake levels of 700 mg/day in adults. Calcium supplementation can be considered if targets cannot be met by dietary intake	2	2
53	Offer smoking cessation advice to all people with SMI who smoke- see section E1 for a full smoking cessation intervention	1	2

Table 3 shows the median value and 75^th^ centile value for the 9 accepted prescribing indicators in the second round of Delphi validation.

**Table 3:** Prescribing indicators accepted in round 2 of Delphi exercise with Median and 75^th^ Centile Value

	**Prescribing Indicator**	**Median Score**	**75**^**th**^** Centile Value**
1	Consider adjunctive metformin, unless contraindicated, in people with SMI (Severe Mental Illness) who are prescribed clozapine or olanzapine and who have demonstrated early weight gain* *early weight gain is defined as 5% of body weight in the first month of treatment and is a predictor of long-term weight gain (> 15% over 3 months)	1	2
2	For people with dementia who have a history of cerebrovascular disease or who have evidence on neurological examination or neuroimaging of cerebrovascular disease consider low-dose aspirin eg. 75 mg daily to prevent or lessen further cognitive decline, unless contraindicated	2	2
3	Perform VTE risk assessment for all adults with SMI admitted to the secondary care setting with reduced mobility	1	2
4	Where gastroprotection is indicated, low dose proton pump inhibitors (PPIs) are the preferred treatment choice in adults with SMI who are prescribed agents with a high anticholinergic cognitive burden (ACB). This is because H_2_ receptor antagonists may increase the ACB and could increase the risk of cognitive impairment	1.5	2
5	Tobacco smoking can alter the pharmacokinetics of psychotropic medications: For people with SMI who are taking TCA’s, mirtazapine, haloperidol and benzodiazepines, do not reduce the dose of the psychotropic drug but instead, monitor for adverse effects	2	2
	Carry out a fracture risk assessment using a validated tool such as QFracture or Frax to determine the need for antiosteoporosis therapy for:		
6	i.Adults over 50 years who are prescribed sodium valproate, carbamazepine, primidone, or phenytoin because these agents are associated with reduced bone mineral density, osteopenia, osteoporosis and increased risk of fractures	2	2
7	ii.Adults < 50 years old on oral steroids (prednisolone > 7.5 mg/day or equivalent for ≥ 3 months)	2	2
8	In individuals with inadequate light exposure or at risk of vitamin D deficiency (eg. nursing home residents, African, African-Caribbean and South Asian populations), supplementation with 10 µg/day of vitamin D should be considered	2	2
9	Adults who consume more than 3.5 units of alcohol per day should be advised to reduce their alcohol intake to nationally recommended levels (< 14 units/week in both men and women)	1	1.75

### Interrater reliability (IRR)

The mean ± SD age of the patients contained in the datasets was 48.3 ± 13.0 years. Male patients accounted for 14 and female accounted for six of the 20 datasets. The total number of underlying physical illnesses was 44 (median 1, IQR 0–3). The total number of prescribed regular medications was 116 (median 4.5, IQR 3–7.5). In preparing the datasets, three instances occurred where HbA1c was missing and two instances where prolactin levels were not available but indicated.

Participants reported an average time of nine minutes (range 5–30 min) to apply the OPTIMISE tool to each dataset, and noted that that the time required for each application reduced following increased familiarity.

The overall Kappa Statistic for OPTIMISE was 0.75 (95% CI 0.71- 0.78). Columns A-D in Table 4 show the level of agreement between the four raters and the standard answers, where, the two pharmacists are labelled rater 1 and 2 and the two physicians are labelled rater 3 and 4. For example, the standard agreed with rater 1 that the OPTIMISE indicators applied in 155 instances across 20 datasets. In 42 instances, the standard did not apply an OPTIMISE indicator but rater 1 did. In 91 instances, the standard applied an OPTIMISE indicator but rater 1 did not. In 1012 instances, both the standard and rater 1 agreed not to apply an OPTIMISE indicator.

**Table 4:** Interrater reliability of OPTIMISE criteria between 4 healthcare professionals across 20 datasets

Rater Combination	A	B	C	D	Ppos	Pneg	Kappa (95% CI)
Standard * rater 1	155	42	91	1012	0.70	0.94	0.639 (0.583 to 0.695)
Standard * rater 2	229	33	28	1010	0.88	0.97	0.848 (0.811 to 0.885)
Standard * rater 3	202	39	44	1015	0.83	0.96	0.790 (0.747 to 0.834)
Standard * rater 4	226	35	20	1019	0.89	0.97	0.865 (0.831 to 0.900)
Standard* raters 1,2,3,4	-	-	-	-	-	-	0.752 (0.717 to 0.782)

A, frequency that both the standard and rater agreed criterion applied; B, standard scored criterion not applied, rater scored criterion applied; C, standard scored criterion applied, rater scored criterion not applied; D, both standard and rater agreed criterion not applied; ppos, proportion of positive agreement; pneg, proportion of negative agreement; CI, confidence interval; IQR, interquartile range

Table 5 shows the level of agreement between physicians and pharmacists when applying OPTIMISE. The Ƙ value among pharmacists (raters 1 and 2) compared to the standard was 0.72 (95%CI 0.68–0.76), indicating substantial agreement and the Ƙ value among physicians (raters 3 and 4) compared to the standard was 0.80 (95% CI 0.77–0.84), indicating substantial agreement.

**Table 5:** Interrater reliability of OPTIMISE between pharmacists and physicians across 20 datasets

Comparators	Kappa (95%CI)
Standard * Pharmacists (raters 1 and 2)	0.72 (0.68 to 0.76)
Standard * Physicians (raters 3 and 4)	0.80 (0.77 to 0.84)

## Discussion

OPTIMISE is a 62-indicator medicines optimisation tool to assist decision making in treating people with SMI. The tool was developed using Delphi metholodogy [52] and IRR was established by four clinicians applying the tool to 20 pseudonymised patient datasets. In a similar format to other medicines optimisation tools [35, 37, 41], the 62 indicators are grouped by body system and make recommendations to optimise medicines through the prompting of monitoring needs and tailored interventions to improve physical health in SMI. The tool has been designed to ensure that the physical health needs of people with SMI are not only equally identified as they would be for people without SMI but that interventions are adapted to account for specific needs of people with SMI and those who are prescribed psychotropic medicines.

Consensus was achieved after two rounds of Delphi validation for the inclusion of 62 prescribing indicators in the final tool. Delphi methodology has proven a successful method for the development and validation of medicines optimisations tools [35, 37, 39, 41]. Typically, up to three rounds of Delphi validation are required to achieve consensus. In this study, consensus was achieved for all except five indicators after two rounds and it was decided that consensus was unlikely to be achieved in a third round for the remaining five indicators.

IRR of OPTIMISE is substantial with an overall level of agreement (Ƙ) of 0.75. This means that when physicians and pharmacists have access to the same medical information relating to people with SMI, they reliably achieve a substantial level of agreement when applying the various indicators within the tool. Validation of other medicines optimisation demonstrated similar levels of agreement in IRR studies. START for example, reported a kappa statistic of 0.68 overall [51]. A high level of IRR improves the utilisation and reliability of OPTIMISE among different healthcare professionals in clinical settings.

OPTIMISE is a clinically important and useful tool that encompasses recommendations to systematically assess and improve cardiovascular, endocrine, respiratory, gastrointestinal and musculoskeletal health. It supports healthcare professionals in knowing how to screen and when to intervene to protect the physical health of people with SMI.

The smoking intervention component of the tool demonstrates how the tool can aid the systematic assessment of physical health needs, prompt health promotion and the implementation of a tailored intervention to improve physical health. For example, one of the cases used in the IRR exercise was a 27 year old male who smokes 20 cigarettes per day and is also prescribed clozapine. By applying OPTIMISE, all raters agreed that they would a) ask and document smoking status, b) opportunistically offer smoking cessation advice and c) offer NRT, varenicline or buproprion to support cessation if he was ready to quit. All raters considered a dose reduction by up to 50% in clozapine and agreed that they would carefully monitor for adverse effects of clozapine and perform a clozapine assay 3–5 days after the clozapine dose adjustment/abrupt cessation of smoking.

A study in an Irish inpatient psychiatric setting revealed 75% of patients (who were current smokers) wanted to quit and 39% had a documented prescription which indicated a potentially clinically significant interaction with smoking or smoking cessation [56]. The same study found that as little as 13% of patients received smoking cessation advice in the previous 12 months. The smoking cessation interventions in OPTIMISE are important and clinically relevant because there is a need to tailor such interventions to the individual and support clinicians in managing these potentially complex scenarios. OPTIMISE has also been designed to align with the ‘Make Every Contact Count’ concept, an evidence based approach to improving people’s health and wellbeing by helping them change their behaviour [57].

OPTIMISE prompts the regular assessment of CV risk in adults with SMI over 40 years using a validated risk assessment tool eg. SCORE, QRISK2, QRISK3. QRISK3 includes SMI and atypical antipsychotic therapy and SCORE includes SMI in their algorithms for risk assessment [58, 59]. Since SMI and atypical antipsychotic therapy are independent risk factors for CVD, using algorithms that exclude these risk factors may underestimate CV risk in people with SMI [60]. The magnitude of this underestimation in people with schizophrenia or bipolar illness could be one-third in men and two-thirds in women [60]. It is therefore important that healthcare professionals are directed towards the most relevant risk assessment tools when assessing CV risk in people with SMI.

Following CV risk assessment, the tool directs the user to consider statin therapy for those adults who have ≥ 10% 10-year risk of developing CVD. In the IRR exercise, this indicator was consistently applied across the raters for four of the 20 datasets. That is, 1 in 5 people were eligible for statin therapy but had not been prescribed lipid lowering therapy nor was there evidence of CV risk assessment. This suggests a high prevalence of prescribing omissions considering the young demographic of the datasets (mean ± SD age of 48.3 ± 13.1 years). The small sample size (*n* = 20) makes it difficult to draw significant conclusions but future research should look to estimate the prevalence of potentially inappropriate prescribing in a larger cohort of people with SMI across different care settings eg. inpatient acute psychiatric services, outpatient clinics and longer-term residential services. In summary, the CV domain in OPTIMISE prompts healthcare professionals to systemically assess people with SMI for CVD and tailor that assessment and the intervention to the needs of the target population.

The inclusion of the VTE indicators in OPTIMISE is important. Epidemiological data suggests that antipsychotic exposure is an independent risk factor for VTE [61, 62]. Uncertainty exists around the implementation of risk reduction strategies for VTE in mental health settings [63]. There is a paucity of evidence for including antipsychotics in risk assessment algorithms but reduced mobility is a recognised risk factor for VTE. OPTIMISE prompts healthcare professionals to consider the impact of reduced mobility on VTE risk in a person with catatonia. One dataset in the IRR exercise was a patient who presented with catatonia. When OPTIMISE is applied, the person is risk assessed using a local VTE risk assessment protocol [64] and pharmacological VTE prophylaxis is indicated. This study highlights the importance of assessing VTE risk in people who present with catatonia due to the significant impact of reduced mobility on VTE risk.

OPTIMISE is primarily designed to prompt clinicians on interventions to improve physical health. There is also some evidence in this study that it also identifies instances where monitoring is not carried out. When applied across 20 datasets in an inpatient setting, OPTIMISE highlighted three instances where HbA1c was not available but should have been checked as part of an annual screen and two instances where prolactin level was not available in people who were prescribed antipsychotics known to raise prolactin levels. There exists a myriad of physical health monitoring guidelines for people with SMI and it is not intended that OPTIMISE replace any of these guidelines [3, 25, 33, 55]. However, a tool designed to improve physical health interventions for people with SMI that incidentally prompts when important investigations are not carried out would be clinically very useful and may improve implementation of screening and intervention strategies.

OPTIMISE consists of 62 indicators. Existing validated screening tools vary in their design and layout. They also vary in length and number of criteria/indicators. The most recent version of STOPP/START contains 114 indicators, PROMPT contains 22 indicators and Beers criteria contains 46 indicators [36, 39, 40]. When applying OPTIMISE as part of the IRR exercise, there was evidence that high familiarity with the tool improved the efficiency of application. For example, one responder cited a 30 min application time for the first dataset but just seven minutes for the last dataset. Another responder quoted an average time of eight minutes for each application of the tool. Utilisation of OPTIMISE in practice requires that it is user friendly and relatively quick to apply. Future research should look at the feasibility of incorporating OPTIMISE into an intervention to improve physical health of people with SMI. This should incorporate behaviour change methodology to understand barriers and facilitators to the implementation to maximise the success of this intervention.

The research team have designed the OPTIMISE tool to be utilised by healthcare professionals who provide care for people with SMI. Since the tool is primarily focused on physical health, the most useful application of the tool would be in the psychiatry setting. The tool could be useful for outpatient, longer term care settings and inpatient/acute psychiatry services.

When introduced in a team-based environment, OPTIMISE could from part of a regular physical health screening and intervention strategy for people with SMI who are utilising mental health services. For example, a multidisciplinary team could elect to use OPTIMISE as part of an admission checklist in the inpatient setting and an annual physical health checklist in the outpatient setting for people with SMI.

Greater awareness and understanding of multimorbidity should influence the design and organisation of health care systems in future [27]. Redesigning information technology systems and embedding screening tools into electronic prescribing systems as demonstrated in SENATOR, OPERAM and PINCER trials is one initiative that could improve management of multimorbidity [43, 44, 65, 66]. Integrating OPTIMISE into a technology-based intervention would allow automatic identification of potentially relevant indicators and faster application of the tool in the clinical setting thus allowing meaningful change in the clinical care for people with SMI.

There are some limitations with this research. The IRR exercise could be expanded to include a larger sample size of both datasets and raters. Expanding the number of datasets and the number of raters would allow a better estimation of reliability and perhaps a larger Kappa could be achieved. The language used in the IRR exercise would be revised in future. We did not offer the participants of the IRR study the opportunity to differentiate between not applying an indicator because it was not applicable, clinical information was not known or they would not apply that indicator in their practice.

Further validation of OPTIMISE is needed including application of the tool to wider cohorts and diverse clinical settings. Improving physical health in people with SMI is complex and the implementation of the OPTIMISE intervention should involve all healthcare professionals involved in the provision of care to people with SMI. Patient and public involvement should be sought for any further research. Further developments for OPTIMISE include the potential adaptation for different populations of people with SMI. Age and gender are factors affecting the prevalence and risk of some physical health co-morbidities [67–69]. Age or gender-specific tools could be utilised within different psychiatry subspecialties and further research could look into the application of an abridged form of the tool in practice with improved efficiency and increased likelihood of eliciting potential prescribing omissions.

## Conclusions

Multimorbidity and poorer physical health contribute to a growing health inequality experienced by people with SMI who are unserved in the area of optimising medicines for physical health. OPTIMISE, a 62 indicator medicines optimisation tool has been developed and validated using Delphi methodology to assist clinicians by prompting consideration for PPOs and tailored interventions to improve physical health.

Interrater reliability of OPTIMISE among physicians and pharmacists was substantial, meaning that physicians and pharmacists can apply OPTIMISE to people with SMI to reliably identify similar interventions.

Further validation of this tool is needed including application of the tool to wider cohorts and diverse clinical settings. Wider application of OPTIMISE would provide an opportunity to improve prescribing quality, prevent avoidable ADEs and improve physical health outcomes as well as patient satisfaction and adherence to treatment.

## Supplementary Information

**Additional file 1.** OPTIMISE (Optimising Physical Health in Mental Illness that is Severe)

## Data Availability

The datasets generated and analysed during the current study are not publicly available due to data protection reasons as dictated by General Data Protection Regulation (GDPR) but anonymised datasets are available from the corresponding author on reasonable request.

## Abbreviations

ABPM: Ambulatory blood pressure monitoring
ACB: Anticholinergic cognitive burden
ADE: Adverse drug event
APA: American psychiatric association
BAP: British association of psychopharmacology
BMI: Body mass index
BTS: British thoracic society
CKD: Chronic kidney disease
COPD: Chronic obstructive pulmonary disease
CV: Cardiovascular
CVD: Cardiovascular disease
DOAC: Direct acting oral anticoagulant
FG: Fasting glucose
GOLD: Global initiative for chronic obstructive lung disease
HbA1c: Glycosylated haemoglobin
HDAT: High dose antipsychotic therapy
IQR: Interquartile range
IRR: Interrater reliability
Ƙ: Cohen’s kappa coefficient
METs: Metabolic syndrome
NICE: National institute for health and care excellence (UK)
NRT: Nicotine replacement therapy
OPTIMISE: Optimising physical health treatment in mental illness that is severe
PIM: Potentially inappropriate medicine
PIP: Potentially inappropriate prescribing
PIPc: Potentially inappropriate prescribing in children
PNeg: Proportion of negative agreement
PPI: Proton pump inhibitor
PPO: Potential prescribing omission
PPos: Proportion of positive agreement
PROMPT: Prescribing optimally in middle-aged people’s treatments
SIGN: Scottish intercollegiate guidelines network
SMI: Severe mental illness
SNRI: Serotonin noradrenaline reuptake inhibitor
SSRI: Selective serotonin reuptake inhibitor
START: Screening tool to alert doctors to right treatment
STOPP: Screening tool of older person’s prescriptions
STOPPFrail: Screening tool of older person’s prescriptions in frail adults with limited life expectancy
TCA: Tricyclic antidepressant
TFTs: Thyroid function tests
TSH: Thyroid stimulating hormone
VTE: Venous thromboembolism
WFSBP: World federation of societies of biological psychiatry
