# Supplementary material for: The development and validation of a medicines optimisation tool to protect the physical health of people with severe mental illness (OPTIMISE)
Source: BMC Psychiatry. 2022 Sep 3;22:585. doi: 10.1186/s12888-022-04235-0 (PMC9441032; doi:10.1186/s12888-022-04235-0)
Supplement: Supplementary file 1 — Additional file 1. OPTIMISE (Optimising Physical Health in Mental Illness that is Severe) [file 12888_2022_4235_MOESM1_ESM.docx]

**OPTIMISE** (**Optimising Physical Health in Mental Illness that is Severe)**

**Before considering the following prescribing criteria, discuss the benefits of lifestyle and diet modification with the individual and explore the potential for switching current psychotropic medicine(s) to alternative agents with a better cardiac and/or metabolic adverse effect profile.**

Lifestyle and Diet Modification recommendations:

- Offer access to a combined healthy eating and physical activity programme to aid in the prevention of weight gain in all adults with psychosis or schizophrenia
- Advise all adults aged 19-64 years to aim to achieve a mixture of moderate and vigorous aerobic activity every week and strength exercise on 2 or more days that work all of the major muscles
- Adults who consume more than 3.5 units of alcohol per day should be advised to reduce their alcohol intake to nationally recommended levels (<14 units/week in both men and women)
- Offer smoking cessation advice to all people with SMI who smoke. See section E1 for a full smoking cessation intervention.

**Section A: Cardiovascular System**

*People with Severe Mental Illness (SMI) have a higher cardiovascular (CV) risk than the rest of the population. Clinicians should be aware that some CV risk assessment tools can underestimate CV risk in people with SMI.*

| **A1** | **Lipid Modification Therapy** |
| --- | --- |
| a | Assess CV risk in all adults with SMI over 40 years using a validated cardiovascular risk assessment tool eg. SCORE, QRISK2, QRISK3 and review on a regular basis eg. annually |
| b | Consider statin therapy (eg. atorvastatin 20mg daily) for those adults who have ≥10% 10-year risk of developing CVD using a validated CV risk assessment tool |
| c | Commence high intensity statin therapy (eg. atorvastatin 80mg daily) in adults with existing CV disease for secondary prevention. Lower doses can be chosen if there are potential drug interactions, high risk of adverse effects or patient preference. |
| d | Commence statin therapy (eg. atorvastatin 20mg daily) for the primary and secondary prevention of CV disease in adults with chronic kidney disease (CKD). Discuss the use of higher doses with a specialist nephrologist/cardiologist if eGFR<30ml/min. |
| e | If total cholesterol > 9mmol/L, non-HDL>7.5mmol/L or TG>10mmol/L refer to a metabolic specialist |
| f | Statins with a high degree of lipophilicity eg simvastatin may be associated with central nervous system disturbance eg. sleep disturbance, nightmares. Consider more hydrophilic statins eg. atorvastatin, pravastatin in people with SMI. |
| **A2** | **Antihypertenstive Therapy** |
| a | Commence antihypertensive therapy in adults <80 years with a clinic blood pressure ≥140mmHg systolic and/or ≥90mmHg diastolic (stage 1 hypertension) and subsequent ambulatory blood pressure monitoring (ABPM) daytime average ≥135/85mmHg who have one or more of the following: |
|  | I. Target organ damage |
|  | II. Established CVD |
|  | III. Renal disease |
|  | IV. Diabetes |
|  | V. A 10 year CVD risk of ≥20% |
| b | Commence antihypertensive therapy to adults of any age with clinic blood pressure ≥160/100mmHg and ABPM ≥150/95mmHg (stage 2 hypertension) |
| **A3** | **Overweight/Obesity** |
|  | *Overweight/obesity is an important risk factor for metabolic syndrome, diabetes and CV disease in SMI*  *Weight gain is a potential adverse effect of psychotropic medicines. Antipsychotic medicines in particular can cause weight gain and while the risk varies between different antipsychotic medicines, significant weight gain can occur within the first few weeks of treatment.* |
| a | Lifestyle interventions should always be part of the first line approach to reducing BMI in overweight/obese individuals with SMI. |
| b | Lifestyle interventions should be continued alongside additional interventions to reduce BMI in overweight /obese individuals with SMI. |
| c | Consider adjunctive metformin, unless contraindicated, in people with SMI who are prescribed clozapine or olanzapine and who have demonstrated early weight gain*.  *Early weight gain is defined as 5% of body weight in the first month of treatment and is a predictor of long-term weight gain (>15% over 3 months) |
| **A4.** | **Smoking** |
| a | Offer smoking cessation advice to all people with SMI who smoke. See smoking E1 for full smoking cessation intervention |
| **A5.** | **Antiplatelet Therapy** |
| a | Commence antiplatelet therapy (aspirin or clopidogrel or prasugrel or ticagrelor) with a documented history of coronary, cerebral or peripheral vascular disease |
| b | For people with dementia who have a history of cerebrovascular disease or who have evidence on neurological examination or neuroimaging of cerebrovascular disease consider low-dose aspirin eg 75mg daily to prevent or lessen further cognitive decline, unless contraindicated |
| **A6.** | **Venous Thromboembolism (VTE) prophylaxis** |
|  | *People taking antipsychotics are at an increased risk of VTE compared with the general population. The absolute increase is low (4 additional cases per 10,000 people treated for 1 year), and may be higher with second generation antipsychotics than first generation antipsychotics. The risk is highest during the first 3 months of therapy.* |
| a | Perform VTE risk assessment for all adults admitted to the secondary care setting with reduced mobility |
| b | For people who present with catatonia, consider the impact of this on mobility when assessing VTE risk and reassess where appropriate |
| c | Commence pharmacological VTE prophylaxis with low molecular weight heparin following risk assessment according to local guidelines |
|  | **Section B: Endocrine System** |
| **B1.** | **Glucose Regulation** |
|  | *People with SMI have higher rates of metabolic syndrome (MetS) and Type 2 Diabetes (T2DM) than the rest of the population.*  *Antipsychotic medications act to increase the risk of diabetes through weight gain and independent mechanisms, both of which affect insulin resistance.* |
| a | If glycosylated haemoglobin (HbA1c) is 42-47mmol/mol or Fasting Plasma Glucose (FG) 5.5-6.9mmol/L: |
|  | i. Offer an intensive structured lifestyle education programme |
|  | ii. If ineffective, consider a trial of metformin |
| b | In existing diabetes, if HBa1c ≥48mmol/mol or FG≥7.0mmol/L refer to an endocrine specialist for optimisation of diabetic control except in older adults where a Hba1c upper limit of 58mmol/L is acceptable |
| **B2.** | **Thyroid Regulation** |
|  | *Some psychotropic medicines including lithium can alter thyroid function tests.*  *Correcting thyroid dysregulation can improve outcomes in affective disorders.* |
| **B2.1** | **Hypothyroidism** |
| a | Consider thyroid supplementation with levothyroxine in adults with SMI if TSH>10mU/L |
| b | When starting levothyroxine, 50-100mcg daily is the recommended starting dose for most adults. In adults >65 years or adults with ischaemic heart disease lower doses of 25mcg can be initiated |
| c | TFT’s should be performed 4-6 weeks after starting levothyroxine and dose adjusted according to response. TSH is the most reliable marker of adequacy of replacement of treatment and a value within the reference range (0.4-4.0mIU/L) should be considered the therapeutic target. |
| d | Adults with subclinical hypothyroidism should have their thyroid function tests repeated within 3-6 months to exclude transient causes of elevated TSH. The measurement of thyroid antibodies in subjects with subclinical hypothyroidism helps to establish the risk of developing overt hypothyroidism |
| e | Do not routinely start thyroid supplementation with levothyroxine for the management of depression in a euthyroid adult or an adult with symptoms that overlap with those of hypothyroidism |
| **B2.2** | **Hyperthyroidism** |
| a | Adults with evidence of hyperthyroidism/ thyrotoxicosis on bloods should be referred to an endocrine specialist |
| **B3.** | **Prolactin** |
| a | Monitor baseline prolactin for all people with SMI before initiating an antipsychotic known to raise prolactin |
| b | Systematically assess for symptoms of hyperprolactinaemia in people with SMI who are prescribed an antipsychotic at 3 months and biannually thereafter |
| c | If symptoms of hyperprolactinaemia appear in a person taking an antipsychotic at any stage, measure prolactin levels |
| d | In symptomatic hyperprolactinaemia, where a dose reduction or a switch to an alternative antipsychotic with a lower potential to elevate prolactin is not possible, consider adjunctive aripiprazole at a dose of 5mg daily. Repeat prolactin levels after at least 1 week to establish benefit. |
| e | Where prolactin levels are > 3000mIU/L, refer to an endocrine specialist to rule out other causes of elevated prolactin |
| f | Dopamine agonists (eg. cabergoline, bromocriptine) should not be initiated in adults with SMI for the management of hyperprolactinaemia except under specialist endocrine advice due to the risk of psychosis |
|  | **Section C: Gastro-intestinal System** |
| **C1.** | **Gastroprotection** |
| a | Consider the increased risk of bleed (not limited to GI bleed) when SSRIs/SNRIs/TCAs combined with aspirin, non-steroidal anti-inflammatory drugs (NSAIDs), warfarin, direct oral anticoagulants (DOACs). |
| b | Where gastroprotection is indicated, proton pump inhibitors (PPIs) are the preferred treatment choice in adults with SMI who are prescribed agents with a high anticholinergic cognitive burden. This is because H2 receptor antagonists may increase the anticholinergic cognitive burden and could increase the long-term risk of cognitive impairment. |
|  | **Section D: Blood and Nutrition** |
| **D1.** | **Anaemia and Vitamin B12 deficiency** |
| a | Identify and manage anaemia and vitamin B12 deficiency as for the general population |
| **D2** | **Folic acid** |
|  | *Some antiepileptic drugs, in particular, enzyme inducing agents eg. Phenytoin, Primodone, Carbamazepine are associated with a reduction in the intestinal absorption and enhanced hepatic metabolism of folic acid.* |
| a | Monitor folic acid levels periodically in people prescribed antiepileptic drugs and correct any folic acid deficiencies |
| b | Always identify and correct vitamin B12 deficiency before starting folic acid |
| c | Consider folic acid 5mg daily for women who are pregnant or planning pregnancy and who are taking antiepileptic drugs including valproate, carbamazepine and possibly lamotrigine. Note that folic acid may reduce the efficacy of lamotrigine and monitor for deterioration in mental illness. |
| d | Do not routinely start folic acid as an augmenting agent in the treatment of depression |
| **D3** | **Omega 3 Fatty Acids** |
| a | Do not routinely prescribed Omega 3 fish oils for people with SMI |
|  | **Section E: Respiratory System** |
| **E1** | **Smoking** |
|  | *People with SMI have substantially higher rates of smoking than the rest of the population*  *People with SMI are equally motivated to quit as the general population* |
| a | Ask and document smoking status for all patients with SMI. |
| b | Opportunistically offer smoking cessation advice to all people with SMI who smoke documenting this advice and current readiness to quit eg. ‘not interested in quiting’, ‘not right time’, ‘would like to but not ready’ etc. For those not ready, check-in again on future interactions. |
| c | For those ready to make a quit attempt, consider nicotine replacement therapy (NRT), varenicline or bupropion to support smoking cessation for people who smoke more than 10 cigarettes per day or who smoke within 30-60minutes of waking with consideration for contraindications and comorbidities. |
| d | When selecting a pharmacological intervention be aware that combinations of different forms of NRT can be used |
| e | Tobacco smoking can alter the pharmacokinetics of psychotropic medicines: |
|  | I. Consider a dose reduction by up to 50% in patients taking clozapine who stop smoking abruptly. Carefully monitor for adverse effects of clozapine and destabilisation of mental illness. Perform a clozapine assay 3-5 days after the dose adjustment or abrupt cessation of smoking. |
|  | II.Consider a dose reduction by up to 20% in patients taking olanzapine who stop smoking abruptly. Carefully monitor for adverse effects of olanzapine and destabilisation of mental illness |
|  | III.For people with SMI who are taking TCA’s, mirtazapine, haloperidol or benzodiazepines, do not reduce the dose of the psychotropic drug but instead, monitor for adverse effects. |
| **E2** | **Asthma and COPD** |
| a | Follow most recent NICE/SIGN/GOLD/BTS guidance for the treatment of Asthma or COPD |
| b | When managing an acute exacerbation of COPD or asthma in adults with SMI consider the potential for steroid induced mania/psychosis. Current guidance recommends prednisolone 40mg/day for 5 days. A lower dose of 30mg/day for the shortest possible duration may be warranted in adults with a history of psychosis/mania depending on the severity of the exacerbation |
| c | Inform adults with SMI who are prescribed systemic corticosteroid therapy of the risk of mania/psychosis at the point of prescribing and monitor for signs of mania/psychosis. |
|  | **Section F: Musculoskeletal System** |
|  | *SMI and psychotropic medication use are associated with increased risk for fracture* |
| **F1** | **Bone Protection** |
| a | Carry out a fracture risk assessment using a validated tool to determine the need for osteoporosis treatment for: |
| I. | Women >/= 65 years and men aged >/= 75 years |
| II. | Adults over 50 years of age with risk factors such as:   1. body mass index <18.5kg/m^2^ 2. history of falls 3. family history of hip fracture 4. oral steroid use (prednisolone >7.5mg/day or equivalent for >/= 3months) 5. secondary causes of osteoporosis 6. smoking 7. >14 units alcohol/week (women) or >21 units alcohol/week (men) |
| III. | Adults < 50 years on oral steroids (prednisolone >7.5mg/day or equivalent for >/= 3 months) |
| IV | Adults > 50 years who are prescribed sodium valproate, carbamazepine, primidone, or phenytoin because these agents are associated with reduced bone mineral density, osteopenia, osteoporosis and increased risk of fractures. |
| b | Adults > 50 years with a history of vertebral fracture should be considered for osteoporosis treatment with an oral bisphosphonate without necessarily requiring risk assessment or DEXA scan |
| c | Adequate dietary calcium consumption is recommended to meet reference intake levels of 700mg/day in adults with SMI. Calcium supplementation can be considered if targets cannot be met by dietary intake. |
| d | In individuals with inadequate light exposure or at risk of vitamin D deficiency (eg. nursing home residents, African, African-Caribbean and South Asian populations), supplementation with 10mcg/day of vitamin D should be considered. |
